# Dissociable Effects of Aging on Salience Subnetwork Connectivity Mediate Age-Related Changes in Executive Function and Affect

**DOI:** 10.3389/fnagi.2018.00410

**Published:** 2018-12-17

**Authors:** Alexandra Touroutoglou, Jiahe Zhang, Joseph M. Andreano, Bradford C. Dickerson, Lisa Feldman Barrett

**Affiliations:** ^1^Department of Neurology, Massachusetts General Hospital, Harvard Medical School, Boston, MA, United States; ^2^Athinoula A. Martinos Center for Biomedical Imaging, Massachusetts General Hospital, Harvard Medical School, Boston, MA, United States; ^3^Department of Psychology, Northeastern University, Boston, MA, United States; ^4^Psychiatric Neuroimaging Division, Department of Psychiatry, Massachusetts General Hospital, Harvard Medical School, Boston, MA, United States; ^5^Frontotemporal Disorders Unit, Department of Neurology, Massachusetts General Hospital, Harvard Medical School, Boston, MA, United States

**Keywords:** resting-state fMRI, salience network subsystems, aging, executive function, arousal

## Abstract

Aging is associated with both changes in affective experience and attention. An intrinsic brain network subserving these functions, the salience network, has not shown clear evidence of a corresponding age-related change. We propose a solution to this discrepancy: that aging differentially affects the connectivity of two dissociated subsystems of the salience network identified in our prior research (Touroutoglou et al., 2012). We examined the age-related changes in intrinsic connectivity between a dorsal and a ventral salience subsystem in a sample of 111 participants ranging in age from 18 years to 81 years old. We predicted that connectivity within the ventral subsystem is relatively preserved with age, while connectivity in the dorsal subsystem declines. Our findings showed that the connectivity within the ventral subsystem was not only preserved but it actually increased with age, whereas the connectivity within the dorsal subsystem decreased with age. Furthermore, age-related increase in arousal experience was partially mediated by age-related increases in ventral salience subsystem, whereas age-related decline in executive function was fully mediated by age-related decreases in dorsal salience subsystem connectivity. These findings explain previously conflicting results on age-related changes in the salience network, and suggest a mechanism for relatively preserved affective function in the elderly.

## Introduction

Substantial evidence indicates that both affect and executive function change as people age, and these changes offer challenges for healthy aging. Elderly people are more easily aroused than are young people and tend to experience arousal as unpleasant (Smith et al., 2005; Gavazzeni et al., 2008; Gruhn and Scheibe, 2008; Moriguchi et al., 2011; Sands and Isaacowitz, 2017). Intense, unpleasant arousal is linked to an increased risk of illness (Cacioppo, 1994; Ong and Allaire, 2005), including cardiovascular disease (Steptoe and Kivimaki, 2012), stroke (Henderson et al., 2013), metastasis of cancer (Garssen, 2004) and metabolic syndrome (Tamashiro, 2011). Age-related decline in attention and executive function (Verhaeghen and Cerella, 2002; Lustig and Jantz, 2015; Hedden et al., 2016) impairs cognitive performance across diverse domains (Wascher et al., 2011; Clapp and Gazzaley, 2012) and increases the risk for injury when walking or driving (Sheridan and Hausdorff, 2007). Understanding the mechanisms of age-related changes in affect and attention may help to identify factors important for healthy aging, as well as shed light on normal brain function throughout the lifespan.

Both affective processing and executive function are linked to the brain’s salience network, a group of structures connected at rest including the anterior insula (AI), dorsal anterior and mid-cingulate cortex (ACC/MCC) and amygdala (Seeley et al., 2007; Touroutoglou et al., 2012).

The strength of connectivity within this network in young adults predicts subjective experiences of anxiety (Seeley et al., 2007), cortisol responsivity (Thomason et al., 2011) and feelings of arousal (Touroutoglou et al., 2012, 2014). Major nodes of the salience network, particularly the AI and ACC/MCC, have also been implicated in various features of executive function, including the orienting of attention (Corbetta and Shulman, 2002), cognitive control (Cole et al., 2013) and performance monitoring (Dosenbach et al., 2007).

This diversity of brain–behavioral relationships within the salience network may be explained by recent evidence suggesting that the salience network is composed of two dissociable subsystems, defined by their connectivity to the dorsal and ventral AI (vAI). These two regions of insula have different patterns of connectivity in macaques (Touroutoglou et al., 2016) and humans (Taylor et al., 2009; Kurth et al., 2010; Cauda et al., 2011; Deen et al., 2011; Kelly et al., 2012; Touroutoglou et al., 2012; Chang et al., 2013; Uddin et al., 2014). In healthy young adults, connectivity within the ventral salience subsystem has been associated with affect whereas connectivity within the dorsal salience subsystem has been associated with executive function (Touroutoglou et al., 2012).

Studies of age-related changes in the integrity of the salience network have shown conflicting results, with decreased age-related connectivity in some cases (Allen et al., 2011; Onoda et al., 2012; He et al., 2013, 2014; Roski et al., 2013; Langner et al., 2015), but preserved or increased connectivity in others (Wang et al., 2012; Cao et al., 2014; Sakaki et al., 2016; Xiao et al., 2018); this is in contrast to clear reductions in connectivity with age in default mode and fronto-parietal networks (Andrews-Hanna et al., 2007; Esposito et al., 2008; Biswal et al., 2010; Wang et al., 2010, 2012; Campbell et al., 2012; Onoda et al., 2012; Betzel et al., 2014; He et al., 2014; Zhang et al., 2014; Shaw et al., 2015; Ward et al., 2015). A closer look at these studies show that those reporting preserved or increased connectivity with age have focused on connectivity to limbic structures within the ventral salience subnetwork such as the amygdala (Wang et al., 2012; Cao et al., 2014; Sakaki et al., 2016; Xiao et al., 2018). In contrast, studies showing decreased salience network connectivity with aging have focused on nodes within the dorsal salience network (Allen et al., 2011; Onoda et al., 2012; He et al., 2013; Roski et al., 2013; Langner et al., 2015). The differential rates of age-related functional connectivity changes are consistent with rates of atrophy. The ventral salience subnetwork contains more agranular cortex, which atrophies more slowly with age when compared to cortex with a granular cytoarchitecture, such as the lateral frontal and parietal regions in the dorsal salience, default mode or fronto-parietal networks (Salat et al., 2004; Fjell et al., 2009, 2014; McGinnis et al., 2011).

In the present study, we hypothesized that the dorsal and ventral salience subnetworks are differentially affected by aging. We predicted that connectivity within the ventral subsystem is relatively preserved with age, while connectivity in the dorsal subsystem declines. We further predicted that age-related changes in stimulus-evoked arousal and executive function are mediated by differences in functional connectivity in the ventral and dorsal salience subsystems, respectively.

## Materials and Methods

### Participants

One hundred eleven adults ranging in age from 18 to 81 (mean age = 46.6, SD = 18.89; 56 females) participated in this study, which involved the collection of resting-state blood oxygenation-level dependent (BOLD) data as well as behavioral and task-evoked data. All participants were right-handed, native English speakers and had normal or corrected-to-normal vision. No participant reported a history of neurological or psychiatric disorders. The protocol of this study was approved by the Institutional Review Board of the Massachusetts General Hospital. All subjects gave written informed consent.

### Behavioral Data Acquisition

#### Affective Experience

Ninety full-color images were selected from the International Affective Picture System (IAPS) to induce affective experiences (Lang et al., 2008). Participants viewed each of the IAPS images on a 120 × 75 cm high definition (Sharp, Aquos) screen placed 2 m from the participant. The images represented five combinations of valence and arousal (i.e., negative valence-high arousal, positive valence-high arousal, negative valence-low arousal, positive valence-low arousal, neutral valence-low arousal). Images were grouped into three blocks of 30 images. Each block contained six images from each of the five valence and arousal categories. To avoid order effects, we randomized the order of the blocks and the order of images within each block during stimulus presentation. For each stimulus, participants viewed the IAPS image for 6 s, then rated the valence and arousal of the image using the Self-Assessment Manikin (SAM; Bradley and Lang, 1994). Only the arousal ratings are reported here, which ranged from “Very calm” (1) to “Very activated” (5). A variable inter-trial interval of 10–15 s followed the rating prior to presentation of the next image. Before beginning the task, participants were familiarized with the SAM rating procedure and practiced by rating five images. The images and rating scales were administered via E-Prime software (Psychology Software Tools, Pittsburgh, PA, USA).

Because the ventral salience network connectivity has been associated with high arousal states such as anxiety (Seeley et al., 2007) or high arousal responses to negative images (Touroutoglou et al., 2012), we focused on high arousal stimuli. For each participant, we obtained a high arousal rating in response to negative evocative images (referred to hereafter as arousal ratings in response to negative images) by calculating the average arousal ratings for all high arousal negative images. In addition, we obtained a high arousal rating in response to positive evocative images (referred to hereafter as arousal ratings in response to positive images) by calculating the average arousal ratings for all high arousal positive images. We removed data for three participants whose arousal ratings were outliers (3 standard deviations below the group mean). We conducted subsequent analyses including arousal ratings with the remaining 108 participants.

#### Executive Function

Executive function, processing speed and set-switching was measured with the Trail Making Test administered before the scans (Reitan, 1958; Strauss et al., 2006). For each participant, the Trail Making Test B (Trails B) score reflected the time in seconds taken to complete the part B of the test, which is thought to require processing speed, visuomotor speed and set-shifting (Strauss et al., 2006). Because the distribution of the Trails B completion time was positively skewed (skewness >1.32), we performed a log transformation on this data. We also inverted the log of Trails B time such that higher Trails B scores indicate better performance. Demographic and neuropsychological characteristics are summarized in Table 1.

**Table 1:** Demographic and neuropsychological characteristics.

Measure	Mean	Minimum	Maximum	Std. Dev.	Std. Error
*N*	111				
Sex (% female)	49.5				
Age (years)	46.65	18.00	81.00	18.90	1.79
Education (years)	16.10	12.00	20.00	2.21	0.21
Trail Making Test B (s)	57.59	19.00	165.00	21.89	2.08
Arousal ratings (5-point scale)	4.58	3.60	5.00	0.40	0.04

*Note: N = 110 for education; N = 108 for arousal ratings. We applied a negative log transformation to Trail Making Test B scores for subsequent analyses*.

### Magnetic Resonance Imaging (MRI) Data Acquisition and Preprocessing

Participants underwent brain imaging on a different day, on average less than 1 week after the behavioral session. Imaging data were collected on a 3T Magnetom Tim Trio system (Siemens Medical Systems, Iselin, NJ, USA) at Massachusetts General Hospital, equipped for echo planar imaging (EPI) with a 12-channel phased-array head coil. Head motion was minimized using head restraints, including a pillow and foam padding. Noise was attenuated with ear plugs. Structural magnetic resonance imaging (MRI) data were acquired using a T1-weighted 3D MPRAGE sequence (TR/TE/flip angle = 2,530 ms/3.48 ms/7°, resolution = 1.0 mm isotropic, FoV = 256 mm, 0% slice gap).

Whole-brain resting state functional MRI (fMRI) data were acquired with echo-planar sequence (TR = 5,000 ms; TE = 30 ms; FA = 90°, FoV = 256 mm, 0% slice gap). These parameters allowed us to obtain 55 slices and have a spatial resolution of 2.0 mm isotropic voxels. The resting state scan was 6.40 min long and the data involved one run of 76 volumes. During all resting-state fMRI (rs-fMRI) runs, participants were directed to keep their eyes open without fixating and to remain as still as possible. Resting state fMRI runs preceded the task-based fMRI runs.

Preprocessing of the resting state fMRI data involved a series of previously established resting state functional connectivity MRI (rs-fcMRI) procedures (Biswal et al., 1995; Vincent et al., 2007; Van Dijk et al., 2010). After removing the first four functional volumes, the following steps were completed: correction for slice-dependent time shifts (SPM2, Wellcome Department of Cognitive Neurology, London, UK), correction for head motion with rigid-body transformation in three translation and three rotations (FMRIB, Oxford, UK), spatial normalization to Montreal Neurological Institute (MNI) atlas space, re-sampling to 2 mm isotropic voxels, spatial smoothing using a 6 mm full width at half-maximum (FWHM) Gaussian kernel, and temporal band-pass filtering to remove frequencies >0.08 Hz. We then removed sources of spurious variance and their temporal derivatives from the data through linear regression (six parameters derived from the rigid-body head motion correction, the signal averaged over the whole brain, the signal averaged over a region within the deep white matter, and the signal averaged over the ventricles) and the residual BOLD time course was retained for functional connectivity analysis.

### Resting State fMRI Analysis

**Figure 1:**
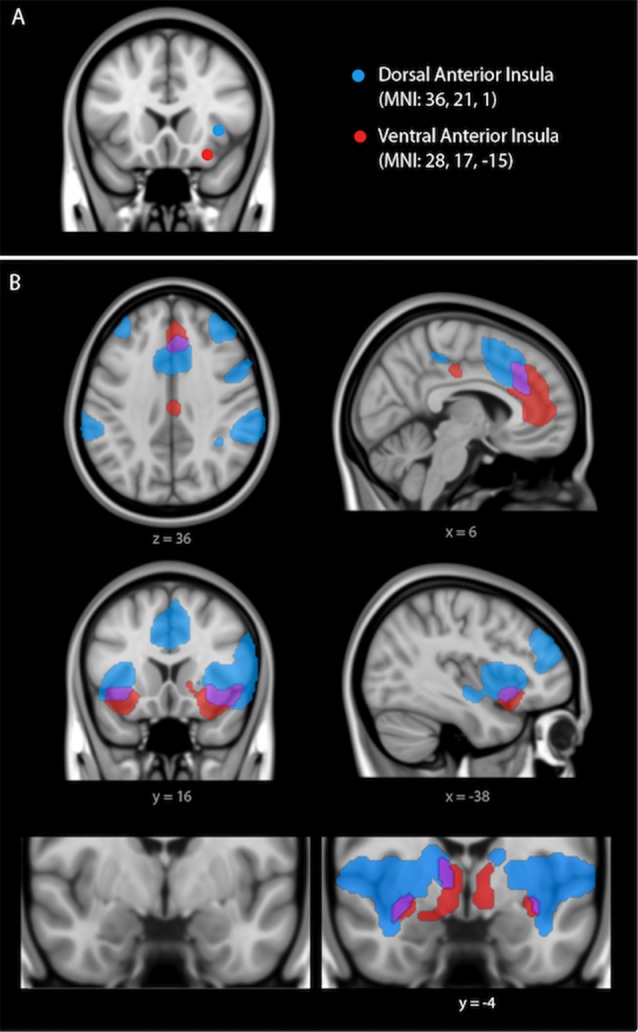
**(A)** Dissociable dorsal and ventral salience networks (right dorsal anterior insula (dAI) seed, blue; right ventral anterior insula (vAI) seed, red) in humans previously published by our laboratory (Touroutoglou et al., 2012). In **(B)**, regions that preferentially correlate with the right dAI seed are shown in blue, regions that preferentially correlate with the right vAI seed are shown in red, and regions that correlate with both seeds are shown in purple. For display purposes, the binarized correlation maps, *z*(r) > 0.2, were overlaid on the 1 mm MNI152 T1-standard template image in FSL (adapted figure from Touroutoglou et al., 2012).

To examine the intrinsic functional connectivity strength within the dorsal and ventral salience subsystems, we used seed-based rs-fcMRI analysis. We took a hypothesis-driven approach and created spherical regions of interest (ROIs; 4-mm radius) around major nodes within each salience subsystem as determined in Touroutoglou et al. (2012; see Figure 1). In that study of young adults, the strength of connectivity within the dorsal salience subsystem predicted individual differences in executive function and the strength of connectivity within the ventral salience subsystem predicted individual differences in the intensity ratings of arousal in response to negative images.

We computed Pearson’s product moment correlations, *r*, for the mean signal time courses of each pair of ROIs. Fisher’s *r*-to-*z* correlation coefficients were then calculated between each ROI pair. To calculate the connectivity within the dorsal salience subsystem, we computed the pairwise connectivity measure of *z*(*r*) values between the dorsal anterior insula (dAI; right dAI coordinates: +36, 21, 1, MNI) and a bilateral region in the mid cingulate (MCC; right MCC coordinates: +4, 16, 46; left MCC coordinates: −2, 14, 46) as determined in Touroutoglou et al. (2012). To calculate the connectivity within the ventral salience subsystem, we computed a pairwise connectivity measure of *z*(*r*) values between the right vAI (right vAI coordinates: +28, 17, −15) and a region in the dorsal amygdala (right amygdala coordinates: +30, −2, −18) found in Gerdes et al. (2010) to predict feelings of arousal during task. This region has been shown to be critical for subjective arousal in a variety of other studies (Phan et al., 2004; Barrett et al., 2007; Wilson-Mendenhall et al., 2013; Touroutoglou et al., 2014). The averaged pairwise connectivity measure of *z*(*r*) values of each pair of ROIs was then used for the analyses of the brain-behavioral relationships with aging.

### Brain-Behavior Relationships

Using a series of linear regression analyses, we first examined the entire sample to determine whether the dissociable relationships between salience subsystem connectivity and behavior that we previously found in an independent sample of young adults (Touroutoglou et al., 2012) would replicate in this sample of adults spanning a broad age range. Specifically, to examine the relationship between ventral salience subsystem connectivity and arousal ratings in response to negative images, we conducted a linear regression analysis using vAI connectivity to amygdala (pairwise connectivity measure of *z*(*r*) values of the right vAI with right amygdala) and arousal ratings as the dependent variable. Additionally, to investigate the possibility that ventral salience connectivity might similarly influence arousal responses to positively valenced stimuli, we repeated this analysis using high arousal, positively valenced stimuli. To examine the relationship between dorsal salience subsystem connectivity and executive function, we conducted a linear regression analysis using dAI connectivity to MCC (averaged pairwise connectivity measure of *z*(*r*) values of the right dAI with left and right MCC) as independent variable and executive function as the dependent variable. We then examined the effects of age on connectivity and behavior. For all these analyses, we controlled for potential effects of sex and education. Brain-behavior analyses were conducted using PASW Statistics 21, Release Version 21.0.0 (SPSS Inc., 2009, Chicago, IL, USA^1^). Results were considered statistically significant at *p* < 0.05.

### Salience Subsystem Connectivity Mediation of the Relationship Between Age and Behavior

We first examined the potential mediating effects of ventral salience subsystem connectivity on the relationship between age and arousal ratings. In Step 1 of our mediation analysis, arousal ratings were regressed on age to examine the total effect of age on subjective arousal (path a). We conducted a linear regression analysis using age as the independent variable and arousal ratings as the dependent variable. Next, we tested whether ventral salience subsystem connectivity mediated the above relationship between age and ratings of arousal. Specifically, in Step 2 of the analysis, the ventral salience subsystem connectivity was regressed on age (path b). We conducted a linear regression analysis using age as the independent variable and ventral salience subsystem connectivity values as the dependent variable. In Step 3 of our meditational analysis, we performed another multiple regression analysis where we regressed the arousal ratings on both age (path a′) and ventral salience subsystem connectivity (path c). In Step 4, we compared the standardized regression coefficients (beta) of the age predictor computed at Step 1 (path a: total effect) and Step 3 (path a′: direct effect) to test the amount of mediation (path bc: indirect effect) by the ventral salience subsystem connectivity predictor (Baron and Kenny, 1986). A Sobel test (Sobel, 1982; Preacher and Hayes, 2008) was conducted to test the significance of mediation. Results were considered statistically significant at *p* < 0.05. We repeated the same analysis procedure to examine whether dorsal salience subsystem connectivity mediated the relationship between age and executive function. Mediation analyses were conducted using PASW Statistics 21, Release Version 21.0.0 (SPSS Inc., 2009, Chicago, IL, USA^1^).

### Data Availability

The data for this study are available on request.

## Results

### Brain-Behavior Relationships

**Figure 2:**
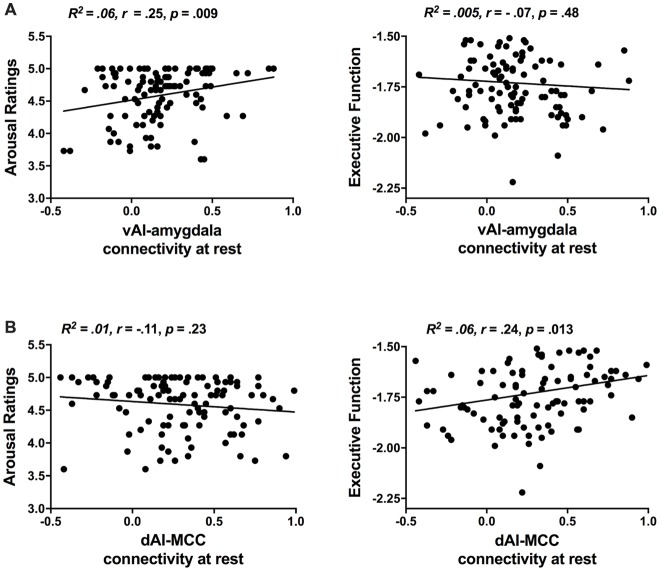
Replication of the dissociable relationships between salience subsystems and behavior previously demonstrated in Touroutoglou et al. (2012). **(A)** Individual differences in ventral salience subsystem connectivity (i.e., strength of vAI intrinsic connectivity to amygdala) are associated with variation in unpleasant arousal (i.e., arousal ratings in response to negative images, but not in executive function performance. **(B)** Individual differences in dorsal salience subsystem connectivity [i.e., strength of dAI intrinsic connectivity to mid-cingulate cortex (MCC)] are associated with variation in performance in executive Trails B task), but not in arousal ratings.

As in our previous study (Touroutoglou et al., 2012), we used rs-fMRI analyses and replicated the dissociable relationships between salience subsystems and behavior in our independent sample including young, middle-aged and older individuals (see Figure 2). As in Touroutoglou et al. (2012), the strength of connectivity within the ventral salience subsystem predicted individual differences arousal ratings in response to negative images (*R*^2^ = 0.06, *r* = 0.25, *p* = 0.000) but not in Trails B performance (*R*^2^ = 0.005, *r* = −0.07, *p* = 0.48), whereas the dorsal salience subsystem predicted individual differences in Trails B performance (*R*^2^ = 0.06, *r* = 0.24, *p* = 0.013) but not in arousal ratings (*R*^2^ = 0.01, *r* = −0.11, *p* = 0.23). Ventral salience connectivity did not predict arousal ratings for high arousal positive images (*R*^2^ = 0.006, *r* = −0.07, *p* = 0.42). As sex and education did not appear to be a confound based on non-significant bivariate correlations with either the independent or dependent variables (*p* > 0.05), these demographic factors were left out of the mediation analysis.

### Aging Effects on Salience Subsystems Connectivity

**Figure 3:**
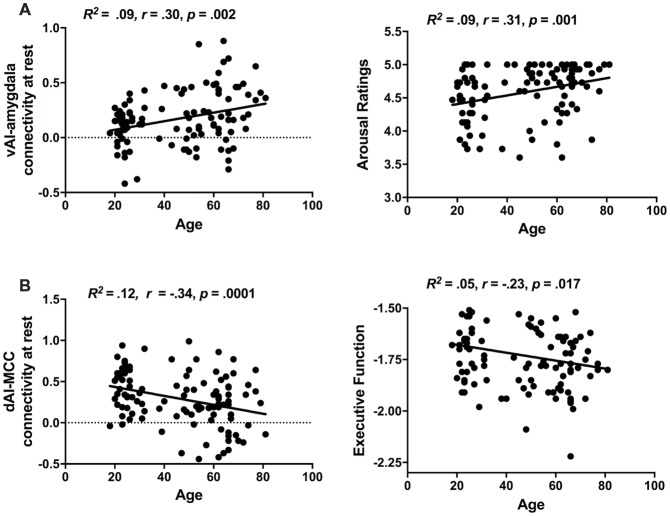
Age-related changes in salience subsystems. **(A)** Older individuals exhibited increased ventral salience subsystem connectivity (i.e., strength of vAI intrinsic connectivity to amygdala) as well as increased unpleasant arousal (i.e., arousal ratings in response to negative images). **(B)** Older individuals exhibit decreased dorsal salience subsystem connectivity (i.e., strength of dAI intrinsic connectivity to MCC) and decreased executive function (i.e., performance in executive Trails B task).

We found that intrinsic connectivity within the ventral salience subsystem was not only preserved as we predicted but it was actually increased with age whereas within the dorsal salience subsystem decreased with age (see Figure 3). The ventral salience subsystem connectivity between the vAI and amygdala was increased with age (*R*^2^ = 0.09, *r* = 0.30, *p* = 0.002). In contrast, the connectivity within the dorsal salience subsystem connectivity between the dAI and MCC was reduced with age (*R*^2^ = 0.12, *r* = −0.34, *p* = 0.0001). Age was also positively correlated with arousal ratings in response to arousing negative images (*R*^2^ = 0.09, *r* = 0.31, *p* = 0.001), such that elderly people expressed more negative arousal than the young, but negatively correlated with executive function (*R*^2^ = 0.05, *r* = −0.23, *p* = 0.017), such that the elderly showed reduced executive function (see Figure 3). No significant correlation was found between age and arousal ratings in response to arousing positive images (*R*^2^ = 0.007, *r* = 0.08, *p* = 0.37). As sex and education did not appear to be a confound based on non-significant bivariate correlations with either the independent or dependent variables, these demographic factors were left out of the mediation analysis.

### Salience Subsystems Connectivity Mediates the Relationship Between Age and Behavior

**Figure 4:**
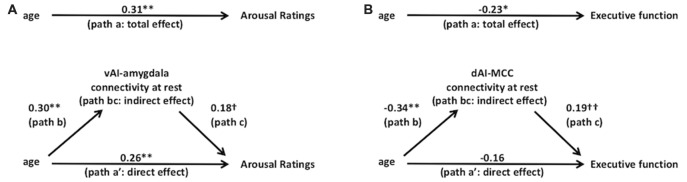
Salience subsystems connectivity mediates the relationships between age and behavior. Solid lines indicate paths and path values indicate standardized beta weights. In **(A)** the upper panel indicates the total effect (unmediated path a, total effect) from age to arousal ratings. In the lower panel, the effect of age on arousal ratings is partially mediated by the ventral salience subsystem connectivity (i.e., vAI connectivity to amygdala at rest). The direct effect of age to arousal ratings is indicated in path a’ and the indirect effect is indicated in the bc path (i.e., path b*path c). In **(B)** the upper panel indicates the total effect (unmediated path a) from age to executive function (i.e., Trails B performance). In the lower panel, the effect of age to executive function is mediated by the dorsal salience subsystem connectivity (i.e., dAI connectivity to MCC at rest). The direct effect of age to executive function is indicated in path a’ and the indirect effect is indicated in the bc path (i.e., path b*path c). **p < 0.01, *p < 0.05, ^†^*p* = 0.05 and ^††^*p* < 0.07.

Using mediation analysis (Baron and Kenny, 1986; Preacher and Hayes, 2008), we found support for our prediction that age-related changes behavior is mediated by salience network connectivity changes. We found that arousal ratings were partially mediated by altered connectivity within the ventral salience subsystem (see Figure 4A). In Step 1 of the mediation model, the regression of arousal ratings on age was significant, *b* = 0.007, *β* = 0.31, *t*_(106)_ = 3.34, *p* = 0.001 (total effect, path a). Step 2 showed that the regression of ventral subsystem connectivity on age was also significant, *b* = 0.004, *β* = 0.30, *t*_(106)_ = 3.21, *p* = 0.002 (path b). Step 3 of the mediation showed that the mediator (vAI-amygdala connectivity within the ventral salience subsystem) controlling for age, marginally predicted affect, *b* = 0.291, *β* = 0.18, *t*_(105)_ = 1.833, *p* = 0.07 (path c). Step 4 of the analyses (direct effect, path a′) revealed that, controlling for the mediator (vAI-amygdala connectivity within the ventral salience subsystem), age was still a significant predictor of arousal ratings, *b* = 0.005, *β* = 0.26, *t*_(105)_ = 2.68, *p* < 0.008. The Sobel test was statistically significant, indicating the indirect effect (path bc, indirect effect) was statistically significant, as was the reduction in path a, indicating significant partial mediation (*z* = 1.66, *p* = 0.048).

As predicted, we found that age-related declines in executive function were mediated by decreased connectivity within the dorsal salience subsystem connectivity (see Figure 4B). Figure 4B shows the standardized beta coefficients for the total as well as the direct and indirect effects. In Step 1 of the mediation model (total effect, path a), the regression of Trails B performance on age, ignoring the mediator, was significant, *b* = −0.002, *β* = −0.23, *t*_(109)_ = −2.43, *p* = 0.017. Step 2 showed that the regression of dorsal subsystem connectivity on age was also significant, *b* = −0.006, *β* = −0.34, *t*_(109)_ = −3.77, *p* = 0.0001 (path b). Step 3 of the mediation showed that the mediator (dAI-MCC connectivity within the dorsal salience subsystem) controlling for age, was closely approaching significance, *b* = 0.097, *β* = 0.19, *t*_(108)_ = 1.95, *p* = 0.053 (path c). Step 4 of the analyses revealed that, controlling for the mediator (dAI-MCC connectivity within dorsal salience subsystem), age was no longer a significant predictor of Trails B performance, *b* = −0.001, *β* = −0.16, *t*_(108)_ = −1.65, *p* = 0.102 (direct effect, path a′). The Sobel test was statistically significant, indicating the indirect effect (path bc, indirect effect) was significant, showing significant mediation (*z* = 1.62, *p* = 0.05).

## Discussion

The results of this study are consistent with our hypothesis that age differentially affects the intrinsic connectivity of the two subsystems of the salience network, and that these differences mediate dissociable age-related influences on the abilities that those networks support. We predicted relative preservation of the circuitry for identifying evocative and affectively important stimuli with age. The results suggest not only preservation but actual increased connectivity between major nodes of the ventral salience subsystem in the aging brain, leading to increased affective reactivity. In contrast, the circuitry for performing a cognitive task requiring set shifting degrades with age, leading to impaired executive function. As we predicted, connectivity in the dorsal salience subsystem declined with age. This result may explain the diverse results reported in previous studies of aging and salience network connectivity (Allen et al., 2011; Onoda et al., 2012; Wang et al., 2012; He et al., 2013, 2014; Roski et al., 2013; Cao et al., 2014; Langner et al., 2015; Sakaki et al., 2016; Xiao et al., 2018). These findings are consistent with age-related atrophy gradients in the human brain (Salat et al., 2004; Fjell et al., 2009, 2014; McGinnis et al., 2011). Studies reporting decreased connectivity of the salience network have not considered connectivity to the amygdala (Allen et al., 2011; Onoda et al., 2012; He et al., 2013; Roski et al., 2013; Langner et al., 2015), which is a node of the ventral, but not dorsal, salience subsystem (Touroutoglou et al., 2012). In contrast, studies reporting preserved salience connectivity have included the amygdala (Wang et al., 2012; Cao et al., 2014). Similarly, increased task-related connectivity between the amygdala and ACC during the presentation of negative stimuli has been reported in the elderly relative to the young (St Jacques et al., 2009, 2010). These findings add to literature suggesting an important protective role of limbic circuitry in successful aging (Harrison et al., 2012, 2018; Rogalski et al., 2013; Sun et al., 2016).

Our brain-behavior relationships replicated the findings of our previous analysis of salience subsystem function (Touroutoglou et al., 2012), suggesting that dorsal salience subsystem connectivity predicts executive function, while ventral salience subsystem connectivity predicts unpleasant arousal in a sample of young, healthy adults. Thus, the functional dissociation of the salience network persists with aging. This finding is consistent with several lines of research showing preserved or enhanced arousal processing in the elderly. Greater ratings of arousal in the elderly relative to the young have been reported for all material, irrespective of valence (Smith et al., 2005; Gavazzeni et al., 2008; Gruhn and Scheibe, 2008; Moriguchi et al., 2011; Sands and Isaacowitz, 2017). Similarly, the tendency to direct attention towards salient arousing stimuli is preserved in aging; both young and elderly people show equivalently enhanced detection speed of high vs. low arousal targets (Mather and Knight, 2006; Leclerc and Kensinger, 2008). Brain imaging studies focused on arousal have also shown equivalent responses for the young and elderly in the amygdala and throughout the salience network (Moriguchi et al., 2011; Kehoe et al., 2013; Dolcos et al., 2014).

Mediation analysis further demonstrated that age-related changes in arousal ratings were partially mediated by increased connectivity within the ventral salience subsystem, such that age-related increases in ventral salience connectivity led to the increased experience of arousal in response to negative evocative images. This suggests that the age-related changes in arousal observed in previous studies may be attributable to age-related increases in ventral salience network connectivity. However, as this mediation was only partial, it seems likely that the experience of arousal is influenced by other brain systems, such as midbrain and brainstem nuclei within the default mode network (Bar et al., 2016), which are important for interoception that serves as the sensory basis for feelings of arousal (Barrett, 2004; Barrett and Bliss-Moreau, 2009; Kleckner et al., 2017). In addition to central nervous systems, the experience of arousal may also be influenced by the peripheral autonomic nervous system (Xia et al., 2017). Furthermore, our participants demonstrated a ceiling effect in their arousal ratings, reducing the available variance for mediation.

While our results showed that arousal experience was positively associated with age, executive function declined in the older members of our sample. This finding is consistent with substantial previous research (Park et al., 2002; Hedden and Gabrieli, 2004; Reuter-Lorenz and Park, 2010). Previous studies, however, have typically related this decline to changes in the connectivity in other brain networks, such as the default mode network (Andrews-Hanna et al., 2007) or fronto-parietal network (Geerligs et al., 2014). The present findings indicate that reduced executive function in older age is also mediated by reduced integrity of the dorsal salience subsystem, possibly through reduced attentional function and speed of processing.

One limitation of this study is that the present analytic approach employed only resting state fMRI. Future research should investigate both the resting state and dynamic coupling of the two salience subsystems to better understand how distinct aspects of salience processing are changed in aging. Additionally, as the strength of brain-behavior correlations reported here are of only moderate strength, further research will be needed to assess the reliability and replicability of these findings. Furthermore, this study used a cross-sectional design to examine mediation models and thus cannot speak to the temporal ordering and causal relationship between brain salience network connectivity and behavior. Future longitudinal studies are needed to elucidate causal models of longitudinal changes in aging (Raz and Lindenberger, 2011). In addition, this study used* a priori* seeds defined in an independent data set of young adults. Most studies of aging have focused on changes in hub connectivity strength with aging, as we did here. It is nonetheless possible that the network topology may also change with aging (Meunier et al., 2009). Future studies should explore this question as well as replicate these findings using an exploratory whole brain approach.

Attention and affective experience are two important psychological phenomena that change with age, but the brain basis underlying these changes remains unclear due to conflicting results across published studies. Here, we show that the brain’s intrinsic salience network has two subsystems that are not affected by aging in a uniform way. The dorsal components of the salience network lose coherence with age, and this decreased connectivity fully mediates an age-related reduction in executive function. The ventral components of the salience network increase their coherence with age, and this increased connectivity partially mediates an age-related increase in arousal-based affective reactivity. These findings resolve conflicting results in prior studies of salience processing in elderly, and enhance our understanding of salience network functions. These findings may also help to resolve conflicting results in clinical studies of disease. Some studies report differences in salience connectivity in age-related diseases such as Alzheimer’s disease (Chand et al., 2017) and minimal hepatic encephalopathy (Chen et al., 2016) while others show no significant differences in Alzheimer’s disease or Mild Cognitive Impairment (Wang et al., 2015). It may be that the dual model of salience network organization suggested here could provide a framework to understand disparate findings on brain network connectivity and disease.

## Author Contributions

AT, JA, BD and LB designed the research. AT, JZ, JA, BD and LB performed the research, analyzed the data and wrote the manuscript.

## Conflict of Interest Statement

The authors declare that the research was conducted in the absence of any commercial or financial relationships that could be construed as a potential conflict of interest.

## Footnotes

^1^www.spss.com

## References

[B1] Allen E. A. Erhardt E. B. Damaraju E. Gruner W. Segall J. M. Silva R. F.. (2011). A baseline for the multivariate comparison of resting-state networks. Front. Syst. Neurosci. 5:2. 10.3389/fnsys.2011.00002 21442040 PMC3051178

[B2] Andrews-Hanna J. R. Snyder A. Z. Vincent J. L. Lustig C. Head D. Raichle M. E.. (2007). Disruption of large-scale brain systems in advanced aging. Neuron 56, 924–935. 10.1016/j.neuron.2007.10.038 18054866 PMC2709284

[B3] Bar K. J. de la Cruz F. Schumann A. Koehler S. Sauer H. Critchley H.. (2016). Functional connectivity and network analysis of midbrain and brainstem nuclei. Neuroimage 134, 53–63. 10.1016/j.neuroimage.2016.03.071 27046112

[B4] Barrett L. F. (2004). Feelings or words? understanding the content in self-report ratings of experienced emotion. J. Pers. Soc. Psychol. 87, 266–281. 10.1037/0022-3514.87.2.266 15301632 PMC1351136

[B5] Barrett L. F. Bliss-Moreau E. (2009). Affect as a psychological primitive. Adv. Exp. Soc. Psychol. 41, 167–218. 10.1016/s0065-2601(08)00404-8 20552040 PMC2884406

[B6] Barrett L. F. Bliss-Moreau E. Duncan S. L. Rauch S. L. Wright C. I. (2007). The amygdala and the experience of affect. Soc. Cogn. Affect. Neurosci. 2, 73–83. 10.1093/scan/nsl042 18392107 PMC2288526

[B102] Baron R. M. Kenny D. A. (1986). The moderator-mediator variable distinction in social psychological research: conceptual, strategic, and statistical considerations. J. Pers. Soc. Psychol. 51, 1173–1182. 10.1037/0022-3514.51.6.1173 3806354

[B7] Betzel R. F. Byrge L. He Y. Goñi J. Zuo X. N. Sporns O. (2014). Changes in structural and functional connectivity among resting-state networks across the human lifespan. Neuroimage 102, 345–357. 10.1016/j.neuroimage.2014.07.067 25109530

[B8] Biswal B. Yetkin F. Z. Haughton V. M. Hyde J. S. (1995). Functional connectivity in the motor cortex of resting human brain using echo-planar MRI. Magn. Reson. Med. 34, 537–541. 10.1002/mrm.1910340409 8524021

[B9] Biswal B. B. Mennes M. Zuo X. N. Gohel S. Kelly C. Smith S. M.. (2010). Toward discovery science of human brain function. Proc. Natl. Acad. Sci. U S A 107, 4734–4739. 10.1073/pnas.0911855107 20176931 PMC2842060

[B101] Bradley M. M. Lang P. J. (1994). Measuring emotion: the self-assessment manikin and the semantic differential. J. Behav. Ther. Exp. Psychiatry 25, 49–59. 10.1016/0005-7916(94)90063-9 7962581

[B10] Cacioppo J. T. (1994). Social neuroscience: autonomic, neuroendocrine and immune responses to stress. Psychophysiology 31, 113–128. 10.1111/j.1469-8986.1994.tb01032.x 8153248

[B11] Campbell K. L. Grady C. L. Ng C. Hasher L. (2012). Age differences in the frontoparietal cognitive control network: implications for distractibility. Neuropsychologia 50, 2212–2223. 10.1016/j.neuropsychologia.2012.05.025 22659108 PMC4898951

[B12] Cao W. Luo C. Zhu B. Zhang D. Dong L. Gong J.. (2014). Resting-state functional connectivity in anterior cingulate cortex in normal aging. Front. Aging Neurosci. 6:280. 10.3389/fnagi.2014.00280 25400578 PMC4212807

[B13] Cauda F. D’Agata F. Sacco K. Duca S. Geminiani G. Vercelli A. (2011). Functional connectivity of the insula in the resting brain. Neuroimage 55, 8–23. 10.1016/j.neuroimage.2010.11.049 21111053

[B14] Chand G. B. Wu J. Hajjar I. Qiu D. (2017). Interactions of insula subdivisions-based networks with default-mode and central-executive networks in mild cognitive impairment. Front. Aging Neurosci. 9:367. 10.3389/fnagi.2017.00367 29170635 PMC5684105

[B15] Chang L. J. Yarkoni T. Khaw M. W. Sanfey A. G. (2013). Decoding the role of the insula in human cognition: functional parcellation and large-scale reverse inference. Cereb. Cortex 23, 739–749. 10.1093/cercor/bhs065 22437053 PMC3563343

[B16] Chen H. J. Chen Q. F. Liu J. Shi H. B. (2016). Aberrant salience network and its functional coupling with default and executive networks in minimal hepatic encephalopathy: a resting-state fMRI study. Sci. Rep. 6:27092. 10.1038/srep27092 27250065 PMC4890427

[B17] Clapp W. C. Gazzaley A. (2012). Distinct mechanisms for the impact of distraction and interruption on working memory in aging. Neurobiol. Aging 33, 134–148. 10.1016/j.neurobiolaging.2010.01.012 20144492 PMC2891289

[B18] Cole M. W. Reynolds J. R. Power J. D. Repovs G. Anticevic A. Braver T. S. (2013). Multi-task connectivity reveals flexible hubs for adaptive task control. Nat. Neurosci. 16, 1348–1355. 10.1038/nn.3470 23892552 PMC3758404

[B19] Corbetta M. Shulman G. L. (2002). Control of goal-directed and stimulus-driven attention in the brain. Nat. Rev. Neurosci. 3, 201–215. 10.1038/nrn755 11994752

[B20] Deen B. Pitskel N. B. Pelphrey K. A. (2011). Three systems of insular functional connectivity identified with cluster analysis. Cereb. Cortex 21, 1498–1506. 10.1093/cercor/bhq186 21097516 PMC3116731

[B21] Dolcos S. Katsumi Y. Dixon R. A. (2014). The role of arousal in the spontaneous regulation of emotions in healthy aging: a fMRI investigation. Front. Psychol. 5:681. 10.3389/fpsyg.2014.00681 25120498 PMC4112914

[B22] Dosenbach N. U. Fair D. A. Miezin F. M. Cohen A. L. Wenger K. K. Dosenbach R. A.. (2007). Distinct brain networks for adaptive and stable task control in humans. Proc. Natl. Acad. Sci. U S A 104, 11073–11078. 10.1073/pnas.0704320104 17576922 PMC1904171

[B23] Esposito F. Aragri A. Pesaresi I. Cirillo S. Tedeschi G. Marciano E.. (2008). Independent component model of the default-mode brain function: combining individual-level and population-level analyses in resting-state fMRI. Magn. Reson. Imaging 26, 905–913. 10.1016/j.mri.2008.01.045 18486388

[B24] Fjell A. M. Westlye L. T. Amlien I. Espeseth T. Reinvang I. Raz N.. (2009). High consistency of regional cortical thinning in aging across multiple samples. Cereb. Cortex 19, 2001–2012. 10.1093/cercor/bhn232 19150922 PMC2733683

[B25] Fjell A. M. Westlye L. T. Grydeland H. Amlien I. Espeseth T. Reinvang I.. (2014). Accelerating cortical thinning: unique to dementia or universal in aging? Cereb. Cortex 24, 919–934. 10.1093/cercor/bhs379 23236213 PMC3948495

[B26] Garssen B. (2004). Psychological factors and cancer development: evidence after 30 years of research. Clin. Psychol. Rev. 24, 315–338. 10.1016/j.cpr.2004.01.002 15245834

[B27] Gavazzeni J. Wiens S. Fischer H. (2008). Age effects to negative arousal differ for self-report and electrodermal activity. Psychophysiology 45, 148–151. 10.1111/j.1469-8986.2007.00596.x 17850240

[B28] Geerligs L. Maurits N. M. Renken R. J. Lorist M. M. (2014). Reduced specificity of functional connectivity in the aging brain during task performance. Hum. Brain Mapp. 35, 319–330. 10.1002/hbm.22175 22915491 PMC6869200

[B29] Gerdes A. B. Wieser M. J. Mühlberger A. Weyers P. Alpers G. W. Plichta M. M.. (2010). Brain activations to emotional pictures are differentially associated with valence and arousal ratings. Front. Hum. Neurosci. 4:175. 10.3389/fnhum.2010.00175 21088708 PMC2982745

[B30] Gruhn D. Scheibe S. (2008). Age-related differences in valence and arousal ratings of pictures from the International Affective Picture System (IAPS): do ratings become more extreme with age? Behav. Res. Methods 40, 512–521. 10.3758/brm.40.2.512 18522062

[B31] Harrison T. M. Maass A. Baker S. L. Jagust W. J. (2018). Brain morphology, cognition, and β-amyloid in older adults with superior memory performance. Neurobiol. Aging 67, 162–170. 10.1016/j.neurobiolaging.2018.03.024 29665578 PMC5955827

[B32] Harrison T. M. Weintraub S. Mesulam M. M. Rogalski E. (2012). Superior memory and higher cortical volumes in unusually successful cognitive aging. J. Int. Neuropsychol. Soc. 18, 1081–1085. 10.1017/s1355617712000847 23158231 PMC3547607

[B33] He X. Qin W. Liu Y. Zhang X. Duan Y. Song J.. (2013). Age-related decrease in functional connectivity of the right fronto-insular cortex with the central executive and default-mode networks in adults from young to middle age. Neurosci. Lett. 544, 74–79. 10.1016/j.neulet.2013.03.044 23583587

[B34] He X. Qin W. Liu Y. Zhang X. Duan Y. Song J.. (2014). Abnormal salience network in normal aging and in amnestic mild cognitive impairment and Alzheimer’s disease. Hum. Brain Mapp. 35, 3446–3464. 10.1002/hbm.22414 24222384 PMC6869630

[B35] Hedden T. Gabrieli J. D. (2004). Insights into the ageing mind: a view from cognitive neuroscience. Nat. Rev. Neurosci. 5, 87–96. 10.1038/nrn1323 14735112

[B36] Hedden T. Schultz A. P. Rieckmann A. Mormino E. C. Johnson K. A. Sperling R. A.. (2016). Multiple brain markers are linked to age-related variation in cognition. Cereb. Cortex 26, 1388–1400. 10.1093/cercor/bhu238 25316342 PMC4785941

[B37] Henderson K. M. Clark C. J. Lewis T. T. Aggarwal N. T. Beck T. Guo H.. (2013). Psychosocial distress and stroke risk in older adults. Stroke 44, 367–372. 10.1161/STROKEAHA.112.679159 23238864 PMC3552144

[B38] Kehoe E. G. Toomey J. M. Balsters J. H. Bokde A. L. (2013). Healthy aging is associated with increased neural processing of positive valence but attenuated processing of emotional arousal: an fMRI study. Neurobiol. Aging 34, 809–821. 10.1016/j.neurobiolaging.2012.07.006 22892310

[B39] Kelly C. Toro R. Di Martino A. Cox C. L. Bellec P. Castellanos F. X.. (2012). A convergent functional architecture of the insula emerges across imaging modalities. Neuroimage 61, 1129–1142. 10.1016/j.neuroimage.2012.03.021 22440648 PMC3376229

[B40] Kleckner I. R. Zhang J. Touroutoglou A. Chanes L. Xia C. Simmons W. K.. (2017). Evidence for a large-scale brain system supporting allostasis and interoception in humans. Nat. Hum. Behav. 1:0069. 10.1038/s41562-017-0069 28983518 PMC5624222

[B41] Kurth F. Zilles K. Fox P. T. Laird A. R. Eickhoff S. B. (2010). A link between the systems: functional differentiation and integration within the human insula revealed by meta-analysis. Brain Struct. Funct. 214, 519–534. 10.1007/s00429-010-0255-z 20512376 PMC4801482

[B100] Lang P. J. Bradley M. M. Cuthbert B. N. (2008). International Affective Picture System (IAPS): Affective Ratings of Pictures and Instruction Manual Technical Report A-8. Gainesville, FL: University of Florida.

[B42] Langner R. Cieslik E. C. Behrwind S. D. Roski C. Caspers S. Amunts K.. (2015). Aging and response conflict solution: behavioural and functional connectivity changes. Brain Struct. Funct. 220, 1739–1757. 10.1007/s00429-014-0758-0 24718622 PMC4193951

[B43] Leclerc C. M. Kensinger E. A. (2008). Age-related differences in medial prefrontal activation in response to emotional images. Cogn. Affect. Behav. Neurosci. 8, 153–164. 10.3758/cabn.8.2.153 18589506

[B44] Lustig C. Jantz T. (2015). Questions of age differences in interference control: when and how, not if? Brain Res. 1612, 59–69. 10.1016/j.brainres.2014.10.024 25451086

[B45] Mather M. Knight M. R. (2006). Angry faces get noticed quickly: threat detection is not impaired among older adults. J. Gerontol. B Psychol. Sci. Soc. Sci. 61, P54–P57. 10.1093/geronb/61.1.p54 16399942

[B46] McGinnis S. M. Brickhouse M. Pascual B. Dickerson B. C. (2011). Age-related changes in the thickness of cortical zones in humans. Brain Topogr. 24, 279–291. 10.1007/s10548-011-0198-6 21842406 PMC3600370

[B47] Meunier D. Achard S. Morcom A. Bullmore E. (2009). Age-related changes in modular organization of human brain functional networks. Neuroimage 44, 715–723. 10.1016/j.neuroimage.2008.09.062 19027073

[B48] Moriguchi Y. Negreira A. Weierich M. Dautoff R. Dickerson B. C. Wright C. I.. (2011). Differential hemodynamic response in affective circuitry with aging: an FMRI study of novelty, valence, and arousal. J. Cogn. Neurosci. 23, 1027–1041. 10.1162/jocn.2010.21527 20521849 PMC3141584

[B49] Ong A. D. Allaire J. C. (2005). Cardiovascular intraindividual variability in later life: the influence of social connectedness and positive emotions. Psychol. Aging 20, 476–485. 10.1037/0882-7974.20.3.476 16248706

[B50] Onoda K. Ishihara M. Yamaguchi S. (2012). Decreased functional connectivity by aging is associated with cognitive decline. J. Cogn. Neurosci. 24, 2186–2198. 10.1162/jocn_a_00269 22784277

[B51] Park D. C. Lautenschlager G. Hedden T. Davidson N. S. Smith A. D. Smith P. K. (2002). Models of visuospatial and verbal memory across the adult life span. Psychol. Aging 17, 299–320. 10.1037/0882-7974.17.2.299 12061414

[B52] Phan K. L. Taylor S. F. Welsh R. C. Ho S. H. Britton J. C. Liberzon I. (2004). Neural correlates of individual ratings of emotional salience: a trial-related fMRI study. Neuroimage 21, 768–780. 10.1016/j.neuroimage.2003.09.072 14980580

[B104] Preacher K. J. Hayes A. F. (2008). Asymptotic and resampling strategies for assessing and comparing indirect effects in multiple mediator models. Behav. Res. Methods. 40, 879–891. 10.3758/BRM.40.3.879 18697684

[B53] Raz N. Lindenberger U. (2011). Only time will tell: cross-sectional studies offer no solution to the age-brain-cognition triangle: comment on salthouse (2011). Psychol. Bull. 137, 790–795. 10.1037/a0024503 21859179 PMC3160731

[B54] Reitan R. M. (1958). Validity of the Trail Making Test as an indication of organic brain damage. Percept. Mot. Skills 8, 271–276. 10.2466/pms.1958.8.3.271

[B55] Reuter-Lorenz P. A. Park D. C. (2010). Human neuroscience and the aging mind: a new look at old problems. J. Gerontol. B Psychol. Sci. Soc. Sci. 65, 405–415. 10.1093/geronb/gbq035 20478901 PMC2883872

[B56] Rogalski E. J. Gefen T. Shi J. Samimi M. Bigio E. Weintraub S.. (2013). Youthful memory capacity in old brains: anatomic and genetic clues from the northwestern superaging project. J. Cogn. Neurosci. 25, 29–36. 10.1162/jocn_a_00300 23198888 PMC3541673

[B57] Roski C. Caspers S. Langner R. Laird A. R. Fox P. T. Zilles K.. (2013). Adult age-dependent differences in resting-state connectivity within and between visual-attention and sensorimotor networks. Front. Aging Neurosci. 5:67. 10.3389/fnagi.2013.00067 24194718 PMC3810651

[B58] Sakaki M. Yoo H. J. Nga L. Lee T. H. Thayer J. F. Mather M. (2016). Heart rate variability is associated with amygdala functional connectivity with mPFC across younger and older adults. Neuroimage 139, 44–52. 10.1016/j.neuroimage.2016.05.076 27261160 PMC5133191

[B59] Salat D. H. Buckner R. L. Snyder A. Z. Greve D. N. Desikan R. S. Busa E.. (2004). Thinning of the cerebral cortex in aging. Cereb. Cortex 14, 721–730. 10.1093/cercor/bhh032 15054051

[B60] Sands M. Isaacowitz D. M. (2017). Situation selection across adulthood: the role of arousal. Cogn. Emot. 31, 791–798. 10.1080/02699931.2016.1152954 26983792

[B61] Seeley W. W. Menon V. Schatzberg A. F. Keller J. Glover G. H. Kenna H.. (2007). Dissociable intrinsic connectivity networks for salience processing and executive control. J. Neurosci. 27, 2349–2356. 10.1523/JNEUROSCI.5587-06.2007 17329432 PMC2680293

[B62] Shaw E. E. Schultz A. P. Sperling R. A. Hedden T. (2015). Functional connectivity in multiple cortical networks is associated with performance across cognitive domains in older adults. Brain Connect. 5, 505–516. 10.1089/brain.2014.0327 25827242 PMC4601675

[B63] Sheridan P. L. Hausdorff J. M. (2007). The role of higher-level cognitive function in gait: executive dysfunction contributes to fall risk in Alzheimer’s disease. Dement. Geriatr. Cogn. Disord. 24, 125–137. 10.1159/000105126 17622760 PMC3163262

[B64] Smith D. P. Hillman C. H. Duley A. R. (2005). Influences of age on emotional reactivity during picture processing. J. Gerontol. B Psychol. Sci. Soc. Sci. 60, P49–P56. 10.1093/geronb/60.1.p49 15643039

[B103] Sobel M. E. (1982). “Asymptotic confidence intervals for indirect effects in structural equations models,” in Sociological Methodology, ed. Leinhart S. (San Francisco, CA: Jossey-Bass), 290–312.

[B65] St Jacques P. Dolcos F. Cabeza R. (2010). Effects of aging on functional connectivity of the amygdala during negative evaluation: a network analysis of fMRI data. Neurobiol. Aging 31, 315–327. 10.1016/j.neurobiolaging.2008.03.012 18455837 PMC3541693

[B66] St Jacques P. L. Dolcos F. Cabeza R. (2009). Effects of aging on functional connectivity of the amygdala for subsequent memory of negative pictures: a network analysis of functional magnetic resonance imaging data. Psychol. Sci. 20, 74–84. 10.1111/j.1467-9280.2008.02258.x 19152542 PMC3633516

[B67] Steptoe A. Kivimaki M. (2012). Stress and cardiovascular disease. Nat. Rev. Cardiol. 9, 360–370. 10.1038/nrcardio.2012.45 22473079

[B68] Strauss E. Sherman E. M. S. Spreen O. (2006). A Compendium of Neuropsychological Tests: Administration, Norms, and Commentary. 3rd Edn. New York, NY: Oxford University Press.

[B69] Sun F. W. Stepanovic M. R. Andreano J. Barrett L. F. Touroutoglou A. Dickerson B. C. (2016). Youthful brains in older adults: preserved neuroanatomy in the default mode and salience networks contributes to youthful memory in superaging. J. Neurosci. 36, 9659–9668. 10.1523/JNEUROSCI.1492-16.2016 27629716 PMC5039247

[B70] Tamashiro K. L. (2011). Metabolic syndrome: links to social stress and socioeconomic status. Ann. N Y Acad. Sci. 1231, 46–55. 10.1111/j.1749-6632.2011.06134.x 21884160

[B71] Taylor K. S. Seminowicz D. A. Davis K. D. (2009). Two systems of resting state connectivity between the insula and cingulate cortex. Hum. Brain Mapp. 30, 2731–2745. 10.1002/hbm.20705 19072897 PMC6871122

[B72] Thomason M. E. Hamilton J. P. Gotlib I. H. (2011). Stress-induced activation of the HPA axis predicts connectivity between subgenual cingulate and salience network during rest in adolescents. J. Child Psychol. Psychiatry 52, 1026–1034. 10.1111/j.1469-7610.2011.02422.x 21644985 PMC3169772

[B73] Touroutoglou A. Bickart K. C. Barrett L. F. Dickerson B. C. (2014). Amygdala task-evoked activity and task-free connectivity independently contribute to feelings of arousal. Hum. Brain Mapp. 35, 5316–5327. 10.1002/hbm.22552 24862171 PMC4335688

[B74] Touroutoglou A. Bliss-Moreau E. Zhang J. Mantini D. Vanduffel W. Dickerson B. C.. (2016). A ventral salience network in the macaque brain. Neuroimage 132, 190–197. 10.1016/j.neuroimage.2016.02.029 26899785 PMC4851897

[B75] Touroutoglou A. Hollenbeck M. Dickerson B. C. Feldman Barrett L. (2012). Dissociable large-scale networks anchored in the right anterior insula subserve affective experience and attention. Neuroimage 60, 1947–1958. 10.1016/j.neuroimage.2012.02.012 22361166 PMC3345941

[B76] Uddin L. Q. Kinnison J. Pessoa L. Anderson M. L. (2014). Beyond the tripartite cognition-emotion-interoception model of the human insular cortex. J. Cogn. Neurosci. 26, 16–27. 10.1162/jocn_a_00462 23937691 PMC4074004

[B77] Van Dijk K. R. Hedden T. Venkataraman A. Evans K. C. Lazar S. W. Buckner R. L. (2010). Intrinsic functional connectivity as a tool for human connectomics: theory, properties, and optimization. J. Neurophysiol. 103, 297–321. 10.1152/jn.00783.2009 19889849 PMC2807224

[B78] Verhaeghen P. Cerella J. (2002). Aging, executive control, and attention: a review of meta-analyses. Neurosci. Biobehav. Rev. 26, 849–857. 10.1016/s0149-7634(02)00071-4 12470697

[B79] Vincent J. L. Patel G. H. Fox M. D. Snyder A. Z. Baker J. T. Van Essen D. C.. (2007). Intrinsic functional architecture in the anaesthetized monkey brain. Nature 447, 83–86. 10.1038/nature05758 17476267

[B80] Wang L. Laviolette P. O’Keefe K. Putcha D. Bakkour A. Van Dijk K. R.. (2010). Intrinsic connectivity between the hippocampus and posteromedial cortex predicts memory performance in cognitively intact older individuals. Neuroimage 51, 910–917. 10.1016/j.neuroimage.2010.02.046 20188183 PMC2856812

[B81] Wang L. Su L. Shen H. Hu D. (2012). Decoding lifespan changes of the human brain using resting-state functional connectivity MRI. PLoS One 7:e44530. 10.1371/journal.pone.0044530 22952990 PMC3431403

[B82] Wang P. Zhou B. Yao H. Zhan Y. Zhang Z. Cui Y.. (2015). Aberrant intra- and inter-network connectivity architectures in Alzheimer’s disease and mild cognitive impairment. Sci. Rep. 5:14824. 10.1038/srep14824 26439278 PMC4594099

[B83] Ward A. M. Mormino E. C. Huijbers W. Schultz A. P. Hedden T. Sperling R. A. (2015). Relationships between default-mode network connectivity, medial temporal lobe structure, and age-related memory deficits. Neurobiol. Aging 36, 265–272. 10.1016/j.neurobiolaging.2014.06.028 25113793 PMC4268379

[B84] Wascher E. Falkenstein M. Wild-Wall N. (2011). Age related strategic differences in processing irrelevant information. Neurosci. Lett. 487, 66–69. 10.1016/j.neulet.2010.09.075 20933055

[B85] Wilson-Mendenhall C. D. Barrett L. F. Barsalou L. (2013). Neural evidence that human emotions share core affective properties. Psychol. Sci. 24, 947–956. 10.1177/0956797612464242 23603916 PMC4015729

[B86] Xia C. Touroutoglou A. Quigley K. S. Feldman Barrett L. Dickerson B. C. (2017). Salience network connectivity modulates skin conductance responses in predicting arousal experience. J. Cogn. Neurosci. 29, 827–836. 10.1162/jocn_a_01087 27991182 PMC5690982

[B87] Xiao T. Zhang S. Lee L. E. Chao H. H. van Dyck C. Li C. R. (2018). Exploring age-related changes in resting state functional connectivity of the amygdala: from young to middle adulthood. Front. Aging Neurosci. 10:209. 10.3389/fnagi.2018.00209 30061823 PMC6055042

[B88] Zhang H. Y. Chen W. X. Jiao Y. Xu Y. Zhang X. R. Wu J. T. (2014). Selective vulnerability related to aging in large-scale resting brain networks. PLoS One 9:e108807. 10.1371/journal.pone.0108807 25271846 PMC4182761

